# A Meta Analysis and Hierarchical Classification of HU-Based Atherosclerotic Plaque Characterization Criteria

**DOI:** 10.1371/journal.pone.0073460

**Published:** 2013-09-03

**Authors:** Wisnumurti Kristanto, Peter M. A. van Ooijen, Marijke C. Jansen-van der Weide, Rozemarijn Vliegenthart, Matthijs Oudkerk

**Affiliations:** Department of Radiology, Center for Medical Imaging – North East, Netherlands, University Medical Center Groningen, University of Groningen, Groningen, The Netherlands; University of Leicester, United Kingdom

## Abstract

**Background:**

Many computed tomography (CT) studies have reported that lipid-rich, presumably rupture-prone atherosclerotic plaques can be characterized according to their Hounsfield Unit (HU) value. However, the published HU-based characterization criteria vary considerably. The present study aims to systematically analyze these values and empirically derive a hierarchical classification of the HU-based criteria which can be referred in clinical situation.

**Material and Methods:**

A systematic search in PubMed and Embase for publications with HU-criteria to characterize lipid-rich and fibrous atherosclerotic plaques resulted in 36 publications, published between 1998 and 2011. The HU-criteria were systematically analyzed based on the characteristics of the reporting study. Significant differences between HU-criteria were checked using Student’s t-test. Subsequently, a hierarchical classification of HU-criteria was developed based on the respective study characteristics.

**Results:**

No correlation was found between HU-criteria and the reported lumen contrast-enhancement. Significant differences were found for HU-criteria when pooled according to the respective study characteristics: examination type, vessel type, CT-vendor, detector-rows, voltage-setting, and collimation-width. The hierarchical classification resulted in 21 and 22 CT attenuation value categories, for lipid-rich and fibrous plaque, respectively. More than 50% of the hierarchically classified HU-criteria were significantly different.

**Conclusion:**

In conclusion, variations in the reported CT attenuation values for lipid-rich and fibrous plaque are so large that generalized values are unreliable for clinical use. The proposed hierarchical classification can be used to determine reference CT attenuation values of lipid-rich and fibrous plaques for the local setting.

## Introduction

Multi-detector-row computed tomography (MDCT) is currently the preferred non-invasive modality to assess the extent of coronary artery disease (CAD) [1], MDCT can reliably exclude the presence of obstructive CAD [2]. Furthermore, contrast-enhanced MDCT shows potential for differentiating types of atherosclerotic plaques, including calcified and non-calcified plaques [3]. MDCT can accurately quantify calcified plaque burden [4–6] and potentially non-calcified plaque volume [3]. However, quantitatively characterizing non-calcified plaque components has been found more challenging [7].

Characterizing the lipid-rich component of non-calcified plaques has become of increasing interest as lipid-rich, thin-capped plaques are considered to have an increased risk of rupture, with the potential sequel of an acute cardiovascular event [8,9]. Early CT studies reported that non-calcified plaque components can be characterized based on their CT attenuation values, expressed in Hounsfield Unit (HU) [10,11]. Since then, a number of studies on this topic has emerged, using new generations of the rapidly evolving MDCT technology [12,13]. However, a reliable and consistent non-calcified plaque characterization based on its HU values is yet to be achieved. The reported plaque-specific HU values vary considerably. Several factors influencing non-calcified plaque HU values have been identified, among others lumen contrast-enhancement and reconstruction kernel [14,15]. However, the fact that each study investigating HU-based non-calcified plaque characterization has different characteristics may also contribute to the considerable variation. Examples of those characteristics are examination type, vessels of interest, and CT-system. The aim of this study is to systematically investigate the published HU-based criteria to characterize non-calcified plaques, and empirically derive a hierarchical classification of the HU-based criteria, in order to assist CT determination of non-calcified components in atherosclerotic plaques in a clinical setting.

## Material and Methods

In this study, we systematically searched and collected publications which reported HU-criteria to characterize lipid-rich and fibrous plaques. Subsequently, the HU-criteria were systematically analyzed based on the specific characteristics of each study.

### Literature Study

**Figure 1:**
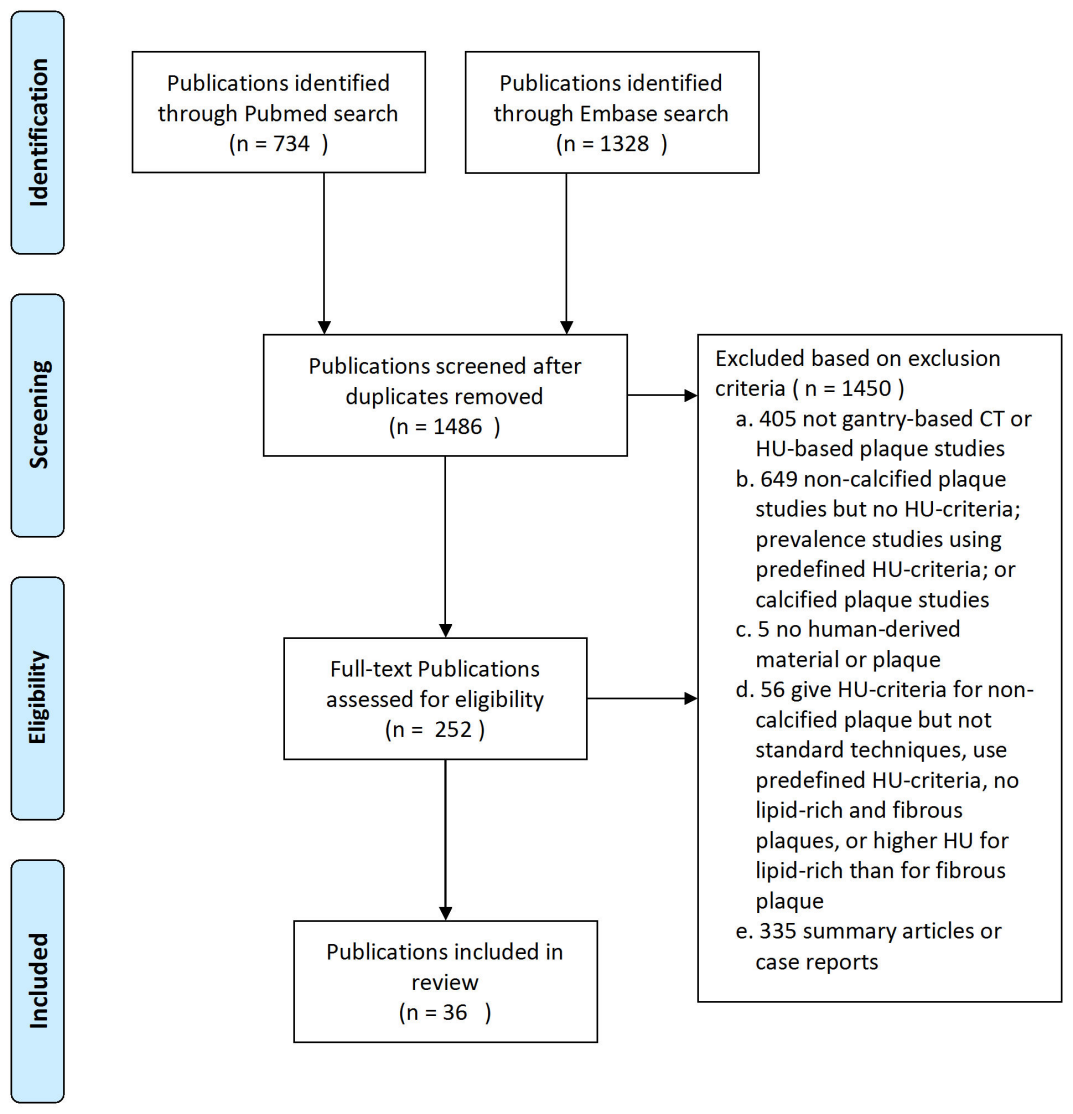
Search strategy and result.

With the guidance of a librarian and using denominator terms for several relevant publications obtained beforehand, a computerized search was performed per April 22^nd^, 2011 to identify relevant publications in Pubmed, using MeSH terms and free text keywords: ("Ultrasonography, Interventional"[Mesh] OR "Coronary Artery Disease"[Mesh] OR "Carotid Artery Diseases"[Mesh]) AND plaque* AND "Tomography, X-Ray Computed"[Mesh] NOT "Review"[Publication Type]; and in Embase, using the keywords: ('endoscopic echography'/exp OR 'coronary artery disease'/exp OR 'carotid artery disease'/exp) AND 'plaque' AND 'computer assisted tomography'/exp NOT 'review'/exp/. Inclusion criteria for publication selection were: 1) original publication; 2) characterization of non-calcified plaques into lipid-rich and fibrous plaques, and report of their specific HU values; 3) using human derived materials; and 4) using other an imaging modality as plaque composition reference. Publications meeting one or more of the defined exclusion criteria were excluded (Figure 1).

The search yielded 2062 publications. After removing 576 duplicates (either overlaps between Pubmed and Embase results or repetitions in each database results), 1486 individual publications were screened by one reviewer (WK) based initially on the title and abstract, and when inclusion was still unclear, on the full-text of the article. In case of doubt about inclusion of a publication, arbitration was performed in a consensus meeting with a second reviewer (PvO). Finally, 1450 out of the remaining 1486 publications were excluded based on the exclusion criteria. No language or publication date related exclusions were made. In total, 36 publications were included in this study [7,10–13,16–46] (Figure 1, Table 1). A preliminary check was performed to evaluate whether one study which used two different CT modalities could be regarded as two separate studies [46] and whether another study which used four different kV settings could be regarded as four separate studies [44]. The preliminary check involved pooling all HU-criteria and pooling all HU-criteria minus one of the aforementioned studies, repeated for all six studies in question. As no significant difference in outcome was found when splitting up these studies, the two studies were treated as six studies resulting in total 40 studies for our systematical analysis obtained from the 36 publications.

**Table 1:** Characteristics of the included publications.

**No**	**Publication**		**Modality**		**Scan Settings**		**Lumen contrast (HU)**	**Subject**					**Reference Modality**	**Plaque**				**Ref#**
	**First Author**	**Year**	**Brand**	**Detector rows**	**Voltage (kV)**	**Collim. - width (mm)**		**Population**			**Study Design**	**Vessel Type**		**Lipid-rich**		**Fibrous**		
								**N**	**Male (%)**	**Age**				**N**	**Value**	**N**	**Value**	
1	Becker	2003	Siemens	4	120	0.5	250	11	45	(34-87)	Ex-vivo	Coronary	Pathology	15	47 + 9	16	104 ± 28	[16]
2	Brodoefel	2008	Siemens	64	120	0.6	NA	13	92	65+7	In-vivo	Coronary	IVUS	NA	(-10-66)	NA	(67-153)	[17]
3	Carrascosa	2003	Philips	4	120	1	NA	30	NA	NA	In-vivo	Coronary	IVUS	105	75.73 + 44.3	14	148.61 + 36.54	[18]
4	Carrascosa	2006	Philips	4	120	1	NA	40	80	52 (33-86)	In-vivo	Coronary	IVUS	188	71.5 + 32.1	45	116.3 + 35.7	[19]
5	Caussin	2004	Siemens	16	NA	NA	NA	21	52	58+13 (39-77)	In-vivo	Coronary	IVUS	12	12 + 38*	4	63.8 + 18.9*	[20]
6	Chopard	2010	Philips	64	120	0.625	NA	21	NA	NA	Ex-vivo	Coronary	Pathology	20	70 + 41	42	83 + 35	[21]
7	de Weert	2005	Siemens	16	140	0.75	0	21	81	64.7 (41-81)	Ex-vivo	Carotid	Pathology	35	45 + 21	28	79 + 20	[22]
8	de Weert	2006	Siemens	16	120	0.75	~400	15	40	70.3 (62-84)	In-vivo	Carotid	Pathology	31	25 + 19	53	88 + 18	[23]
9	Estes	1998	Siemens	1	NA	3	150-300	20	80	74 (57-85)	In-vivo	Carotid	Pathology	NA	39 + 12	NA	90 + 24	[10]
10	Ferencik	2006	Siemens	16	120	0.75	250	6	67	77+1	Ex-vivo	Coronary	OCT	41	29 + 43	40	101 + 21	[24]
11	Galonska	2008	GE	16	120	0.625	308	30	67	61.5+13.4	Ex-vivo	Coronary	Pathology	33	(26-67) median: 44	21	(37-124) median: 67	[25]
12	Hur	2009	Siemens	64	120	0.6	NA	39	72	59 (45-74)	In-vivo	Coronary	IVUS	10	54 + 13	11	82 + 17	[26]
13	Iriart	2007	Siemens	16	120	0.75	NA	20	85	53+12 (38-83)	In-vivo	Coronary	IVUS	NA	38 + 33	NA	94 + 44	[27]
14	Jin	2006	Siemens	16	120	NA	NA	49	55	NA	In-vivo	Carotid	DUS	NA	6 + 28	NA	51 + 19	[28]
15	Kim	2009	GE	64	120	0.625	NA	42	48	66+9	In-vivo	Coronary	IVUS	28	52.9 + 24.6	43	98.6 + 34.9	[29]
16	Kitagawa	2007	GE	64	120	0.625	~350	21	76	66+9	In-vivo	Coronary	IVUS	25	18 + 17	13	67 + 21	[30]
17	Kopp	2001	Siemens	4	120	1	NA	6	67	60+8	In-vivo	Coronary	IVUS	2	0.5 + 7.8*	2	67 + 22.6*	[11]
18	Leber	2004	Siemens	16	120	0.75	NA	37	NA	NA	In-vivo	Coronary	IVUS	62	49 + 22	87	91 + 22	[31]
19	Leschka	2010	Siemens	64	120	0.6	300	25	72	72+13 (38-85)	Ex-vivo	Coronary	Pathology	91	40 + 17*	43	91 + 16	[32]
20	Marwan	2011	Siemens	64	120	0.6	NA	40	75	59+10 (52-85)	In-vivo	Coronary	IVUS	15	67 + 31	40	96 + 40	[33]
21	Motoyama	2007	Toshiba	16	135	0.5	258+43 (174-384)	37	84	66+12	In-vivo	Coronary	IVUS	18	10.6 + 11.6	40	78.1 + 20.8	[34]
22	Nikolaou	2004	Siemens	4	120	0.5	250	17	65	(38-86)	Ex-vivo	Coronary	Pathology	16	45 + 16	21	97 + 31	[36]
23	Nikolaou	2004	Siemens	4	120	0.5	242+28	13	62	(34-87)	Ex-vivo	Coronary	Pathology	10	47 + 13	11	87 + 29	[35]
24	Pohle	2007	Siemens	16	120	0.75	NA	32	72	59+8	In-vivo	Coronary	IVUS	84	58 + 43	42	121 + 34	[12]
25	Qiu	2006	Philips	64	120-140	0.625	NA	6	67	77.5+9.3	In-vivo	Coronary	IVUS	2	-21.5 + 36.6	4	85.3 + 14.3	[37]
26	Sakakura	2006	Toshiba	16	135	0.5	NA	16	69	63+12 (42-80)	In-vivo	Coronary	IVUS	6	50.6 + 14.8	11	131 + 21	[38]
27	Schroeder	2001	Siemens	4	140	1	NA	15	87	58+10 (44-71)	In-vivo	Coronary	IVUS	12	14 + 26	5	91 + 21	[39]
28	Schroeder	2004	Siemens	4	140	1	182+34	12	NA	63+17	Ex-vivo	Coronary	Pathology	6	42 + 22	6	70 + 21	[41]
29	Schroeder	2004	Siemens	16	120	0.75	237+17	9	NA	NA	Ex-vivo	Popliteal	Pathology	13	51 + 20*	18	126 + 99	[40]
30	Shen	2010	GE	64	120	0.6	NA	91	58.2	64.78+9.19 (38-79)	In-vivo	Coronary	IVUS	6	52.52 + 15.71	36	108.32 + 43.44	[42]
31	Soeda	2011	Siemens	64	120	0.6	NA	17	82.4	63.5+8.4	In-vivo	Coronary	OCT	78	28.9 + 30.6	42	77.5 + 25.7	[43]
32	Sun	2008	Toshiba	64	120-135	0.5	398+74	26	65	56	In-vivo	Coronary	IVUS	NA	79 + 34	NA	90 + 27	[13]
33	Tanami	2010	GE	32	80	0.625	0	15	73.3	72+9	Ex-vivo	Coronary	Pathology	39	20.5 + 6.5	30	28.1 + 4.3	[44]
					100										21.8 + 7.3		27.8 + 4.7	
					120										23.1 + 7.2		27.1 + 5	
					140										23.9 + 7.2		27.3 + 5.1	
34	Wintermark	2008	GE	16	120	0.625	NA	8	100	61 (55-69)	In-vivo	Carotid	Pathology	NA	32.6 + 20	NA	46.4 + 19.9	[7]
35	Wu	2007	GE	16	120	1.25	NA	30	73	58 (43-75)	In-vivo	Coronary	IVUS	16	23 + 18	19	69 + 21	[45]
36	Xiao	2007	GE	16	120	0.625	NA	25	NA	(50-72)	Ex-vivo	Coronary	Pathology	13	53 + 12	10	106 + 17	[46]
			Toshiba	64	120	0.5									51 + 13		110 + 19	

Notes:1. Values in the columns Lumen Contrast, Age, and Plaque Values are in means, with the range in brackets.

2. *: Values were self-calculated

3. NA: data were not available

HU-criteria were collected for lipid-rich plaques (synonyms used: soft, hypoechoic, lipid, lipid-rich, hypodense, or lipid-rich necrotic core) and for fibrous plaques (synonyms used: intermediate, hyperechoic, fibrous, fibrous-rich, or connective tissue), as has been characterized by each study based on each chosen reference modality. When only the raw or partial data were presented in the publications, the plaque value (mean + standard deviation [SD]) was calculated [11,20,32,40].

### Systematic Analysis of Published HU-criteria

First, all published HU-criteria were pooled. Next, the correlation between published HU-criteria and the reported lumen contrast-enhancement was investigated. Finally, the published HU-criteria were pooled based on similarity of the studies concerning: 1) examination type (in-vivo or ex-vivo), 2) vessel type (coronary or other arteries), 3) CT-system brand, 4) detector-rows, 5) voltage-setting, and 6) collimation-width. Studies using a dual-source CT (DSCT) [26,32,33,43] were grouped with 64-row MDCT studies because of the similarity in number of detector-rows. For the remainder of this article, DSCT was regarded as equal to 64-row MDCT. Pooling was performed by the pooled statistics, using the following formulas:

$$\begin{array}{c} mean_{pooled}=\frac{N_{1}mean_{1}+N_{2}mean_{2}+...+N_{k}mean_{k}}{N_{1}+N_{2}+...+N_{k}} \end{array}$$ (1)

$$stdev_{pooled}=\sqrt{\frac{(N_{1}-1)stdev_{1}{}^{2}+(N_{2}-1)stdev_{2}{}^{2}+...+(N_{k}-1)stdev_{k}{}^{2}}{N_{1}+N_{2}+...+N_{k}-k}}$$ (2)

Note:

N amount of plaques region of interests (ROIs), segments, or squares used to make the mean + SD
k number of studies included

Not all information to compute the pooled statistics was available in 8 studies. Contact information of corresponding authors was used to contact them in 7 of these studies. Of these, one author replied but was not able to provide the requested missing information. Only those studies providing all the necessary information for pooling (table 1) were included in each pooling calculation.

### Hierarchical Classification

The analysis was extended by systematically classifying the HU-criteria by the following hierarchy: examination type, vessel type, CT-system brand, detector-rows, voltage-setting, and collimation width. Comparisons were made between criteria at the lowest tree branches. HU-criteria which were not significantly different were pooled.

### Statistical Analysis

The correlation between the published HU-criteria and the reported lumen contrast-enhancement was analyzed using linear regression analysis and was expressed as the coefficient of determination (r^2^), ranging from 0 to 1 with r^2^ = 1 indicating perfect correlation. Significant differences between the pooled HU-criteria were determined using one way analysis of variance (ANOVA) test when more than 2 groups are compared or unpaired student’s t-test with unequal variances assumed when 2 groups are compared in Prism 6 (GraphPad Software, Inc., USA) at p value < 0.05.

## Results

Preliminary analysis on the 40 analyzed studies showed that:

1. Sixteen were ex-vivo studies and 24 in-vivo studies;
2. In 34 coronary arteries were studied and in 6 other arteries (i.e. carotid and popliteal arteries);
3. Eleven studies were performed on General Electric (GE) CT-systems, 4 on Philips systems, 21 on Siemens systems, and 4 on Toshiba systems;
4. One study was performed on a 1 detector-row CT-system, 8 on 4-row MDCT, 15 on a 16-row MDCT, 4 on a 32-row MDCT, and 12 on a 64-row MDCT;
5. Two used the voltage setting <120 kV, 28 studies used 120kV, 6 studies used >120kV, and 2 studies used variable kV settings. In 2 studies, the kV setting was not reported;
6. The collimation width applied in the CT-system in 7 studies was <0.6 mm, 17 studies applied 0.6-0.7 mm, 7 studies applied 0.7-0.8 mm, and 7 studies applied >0.8 mm collimation width In 2 studies, the collimation width was not reported.
7. Thirty eight studies reported the plaque HU values in mean + SD format, 1 study reported plaque median HU value and the range, and 1 study only HU value range.
8. Out of 20 in-vivo studies that examined the coronaries, 19 studies used intra-vascular ultrasound (IVUS) and 1 study used optical coherence tomography (OCT) as plaque composition reference. Out of 4 in-vivo study that examined the carotid arteries, 3 used histopathology and 1 used Doppler ultrasound (DUS) as plaque composition reference.
9. Out of 14 ex-vivo studies that examined coronaries, 13 studies used histopathology and 1 study used OCT as plaque composition reference. One ex-vivo study that examined carotid arteries and another that examined popliteal arteries used histopathology as plaque composition reference.

### Systematic Analysis of Published HU-criteria

**Figure 2:**
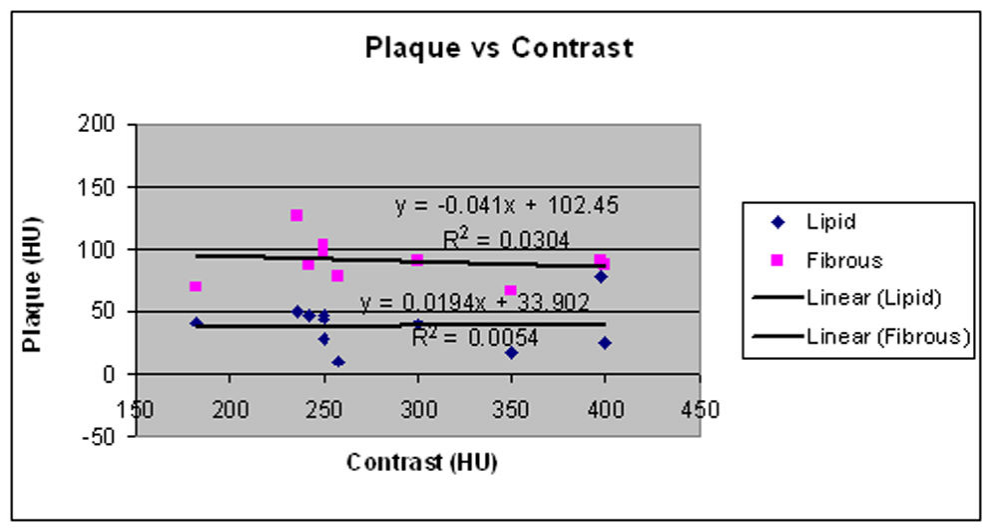
HU criteria for lipid-rich and fibrous plaques versus the reported lumen contrast-enhancement.

Pooling all published HU-criteria, the values for lipid-rich and fibrous plaques were: 47 + 29 HU and 86 + 29 HU, respectively. The published mean HU-criteria showed a low correlation with lumen contrast-enhancement, for lipid-rich (r^2^ = 0.0054 ; p > 0.05) and fibrous plaques (r^2^ = 0.0304; p < 0.05) (Figure 2).

Results of the pooled HU-criteria based on similar study characteristics are shown in Table 2. The HU-criteria in all study characteristic groups were significantly different, except the HU-criteria for fibrous plaque between coronary and other arteries. Comparison between HU-criteria within one group of study characteristics shows all were significantly different except 12 pair of HU-criteria (see table 2).

**Table 2:** Pooled HU-criteria.

**Characteristics**		**Ns**	**Lipid**			**Fibrous**		
			**Np**	**Mean + st dev**	**Sig. Diff.**	**Np**	**Mean + st dev**	**Sig. Diff.**
Study	Ex-vivo	15	429	36+20	Yes	365	73+30	Yes
design	In-vivo	18	701	53+33		511	96+29	
Vessel	Coronary	30	1051	47+29	Yes	777	85+27^‡^	No
Type	Other Arteries	3	79	38+20		99	92+45^‡^	
Brands	GE	9	244	28+13*	Yes	241	61+24^§^	Yes
	Philips	4	315	72+37		105	106+35	
	Siemens	17	534	41+29^††^		469	94+32^║^	
	Toshiba	3	37	31+13*,^†^^†^		61	93+21^§^,||	
Rows	4	8	354	67+35	Yes	120	108+32	Yes
	16	11	332	42+31^†^		352	95+31^#^	
	32	4	156	22+7		120	28+5	
	64	9	288	41+25^†^		284	91+32^#^	
Voltage	<120	2	78	21+7^‡‡^	Yes	60	28+5	Yes
(kV)	120	23	921	52+31		688	94+32	
	>120	6	116	29+17^‡‡^		120	71+18	
Collimation	<0.6	6	78	39+13^§§,║║^	Yes	109	95+25**	Yes
width	0.6-0.7	14	444	34+21^§§^		404	72+27	
(mm)	0.7-0.8	6	266	46+33^║║^		268	98+34**,^##^	
	>0.8	6	329	67+36		91	106+32^##^	

Note:1. *Ns*: total amount of studies included in the pooling calculation. There were studies excluded because of incomplete data needed for pooling calculation or unclear characteristics needed for classification.

2. *Np* : total amount of plaques ROIs, segments, or squares of the studies of similar characteristic used to make the mean+st dev

3. All comparisons between groups’ HU-criteria within one type of characteristics were significantly different (p≤0.05) except the 12 pairs marked with the same symbols (*, †, ‡, §, ||, #, **, † †, ‡ ‡, § §, ||||, and # #).

### Hierarchical classification of the published HU-criteria

**Figure 3:**
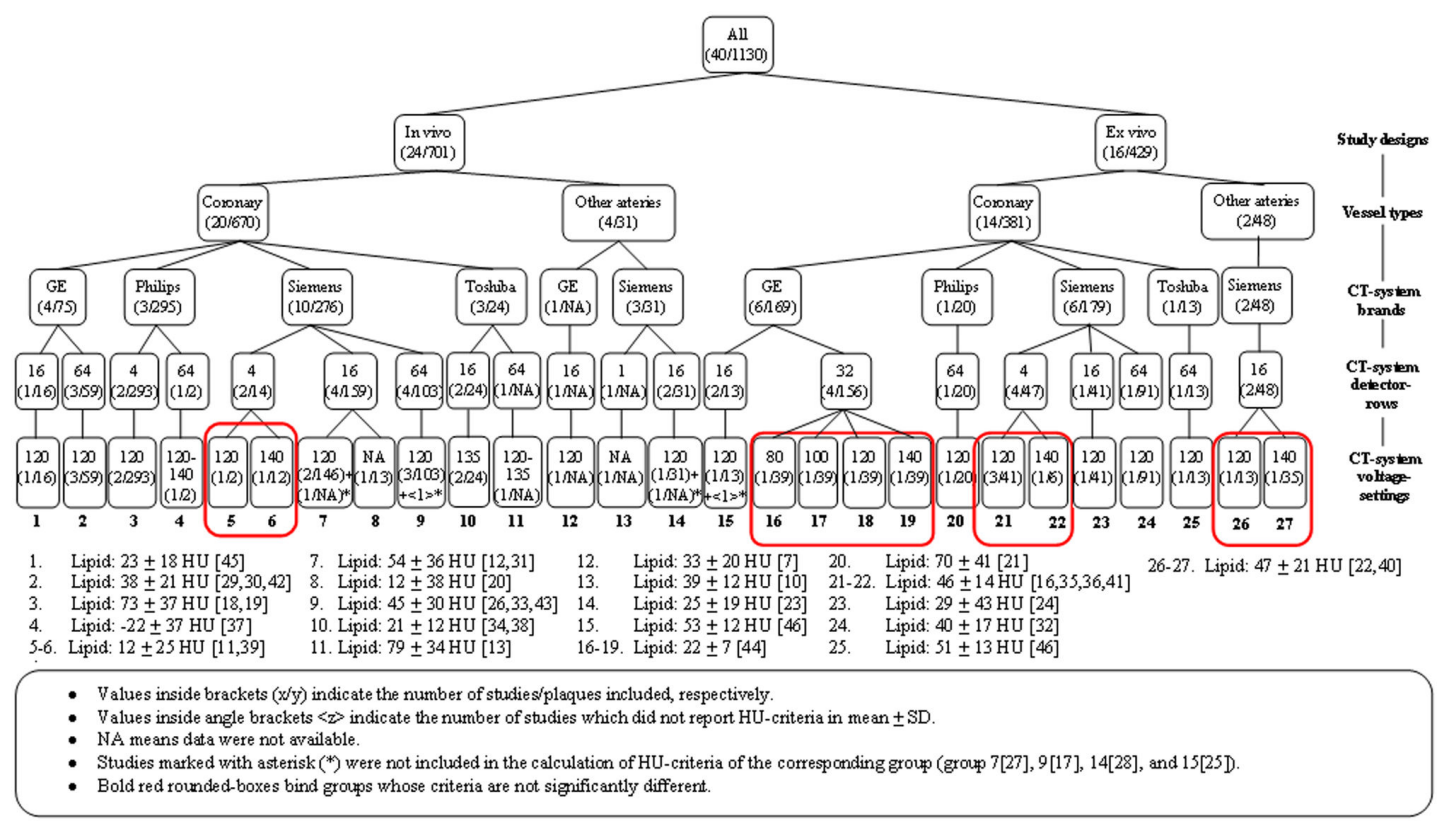
Hierarchical classification of HU-criteria for lipid-rich plaques.

**Figure 4:**
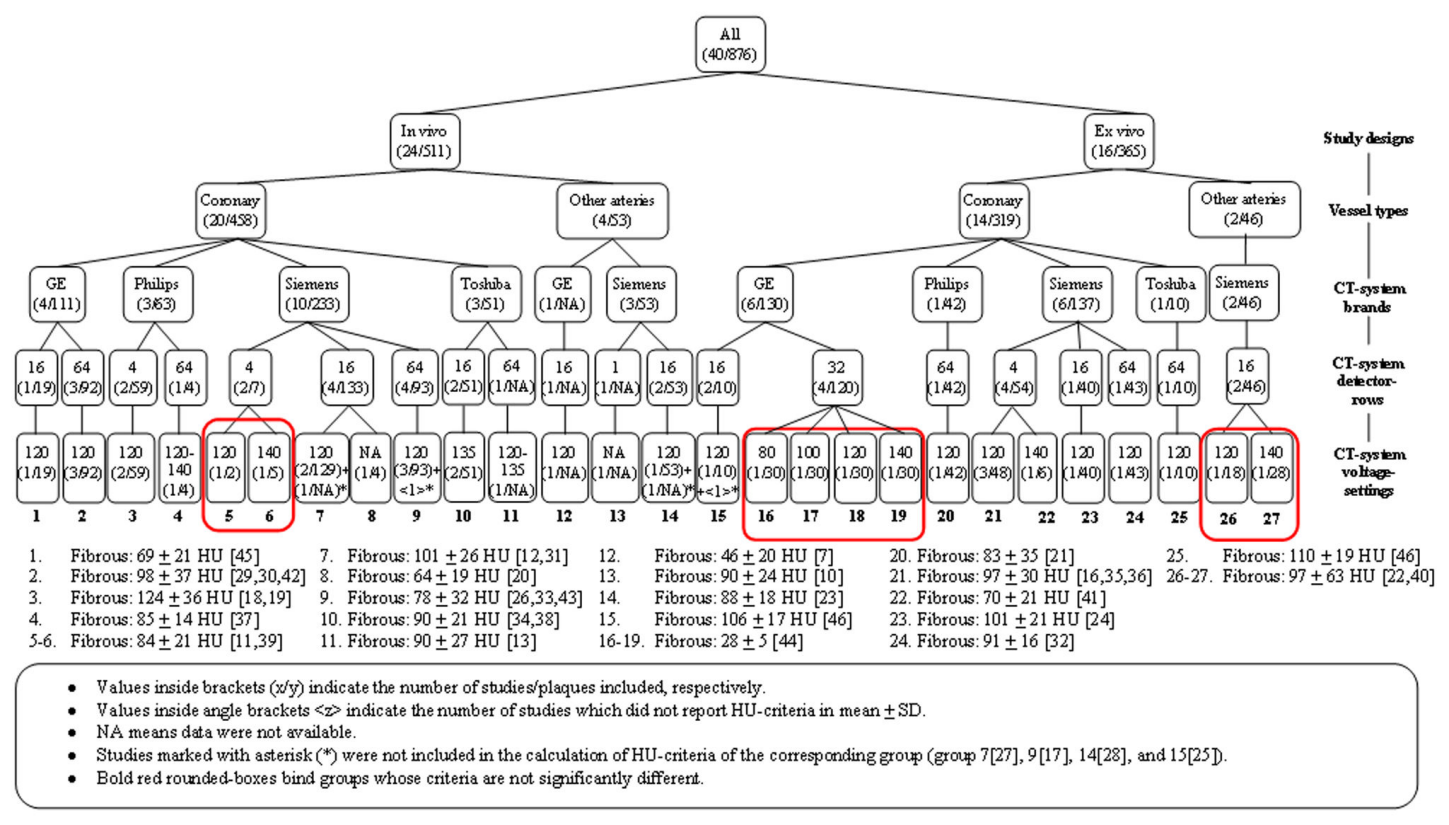
Hierarchical classification of HU-criteria for fibrous plaques.

**Figure 5:**
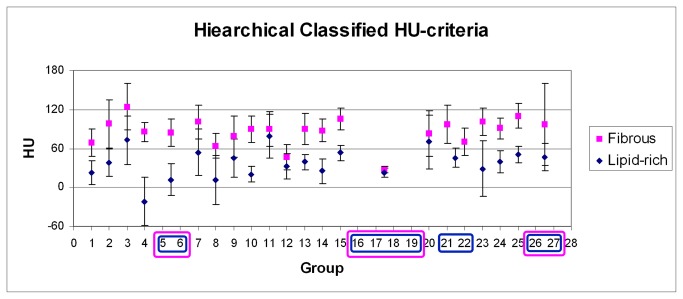
The hierarchically grouped HU-criteria for lipid-rich and fibrous plaques along with their +/-1 standard deviation. The blue and purple rounded-boxes bound the groups that give non-significant different HU-criteria and therefore pooled, for lipid-rich and fibrous plaque, respectively.

Extending the analysis, a hierarchical classification of the published HU-criteria was performed, resulting in 27 distinct HU-criteria groupings (Figures 3 and 4). No further classification based on collimation-width was performed because the studies included in each of these 27 groups had the same collimation-width or did not provide the collimation-width information. The criteria at the lowest tree branches, which were not significantly different, were pooled (boxed groups in Figures 3 and 4), resulting in 21 and 22 distinct HU-criteria groupings for lipid-rich and fibrous plaques, respectively. Comparing the HU-criteria for lipid-rich plaque of each group to each other, 34% (52 out of 153 comparisons) were significantly different, of which 51.9% (27 out of 52 comparisons) were significantly different at p < 0.001 (Table 3). For fibrous plaque, 22.2% (38 out of 171 comparisons) of the HU-criteria were significantly different, of which 57.9% (22 out of 38 comparisons) were significantly different at p < 0.001 (Table 4). A visual representation of the hierarchically grouped HU-criteria along with their range (+/- 1 standard deviation) is given in Figure 5.

**Table 3:** Comparison of HU-criteria for lipid-rich plaque between hierarchical-classified groups.

		**Group**																				
		**1**	**2**	**3**	**4**	**5-6**	**7**	**8**	**9**	**10**	**11**	**12**	**13**	**14**	**15**	**16-19**	**20**	**21-22**	**23**	**24**	**25**	**26-27**
**Group**	**1**	-	*	1	*	*	2	*	*	*	NA	NA	NA	*	*	*	2	*	*	*	*	*
	**2**	*	-	1	*	*	2	*	*	*	NA	NA	NA	*	*	2	2	*	*	*	*	*
	**3**	1	1	-	2	1	1	1	1	1	NA	NA	NA	1	*	1	*	1	1	1	*	1
	**4**	*	*	2	-	*	2	*	*	*	NA	NA	NA	*	*	*	2	*	*	*	*	*
	**5-6**	*	*	1	*	-	1	*	2	*	NA	NA	NA	*	2	*	1	2	*	*	2	2
	**7**	2	2	1	2	1	-	1	*	1	NA	NA	NA	1	*	1	*	*	2	2	*	*
	**8**	*	*	1	*	*	1	-	2	*	NA	NA	NA	*	2	*	1	2	*	*	*	2
	**9**	*	*	1	*	2	*	2	-	2	NA	NA	NA	*	*	1	2	*	*	*	*	*
	**10**	*	*	1	*	*	1	*	2	-	NA	NA	NA	*	*	*	1	*	*	*	*	2
	**11**	NA	NA	NA	NA	NA	NA	NA	NA	NA	-	NA	NA	NA	NA	NA	NA	NA	NA	NA	NA	NA
	**12**	NA	NA	NA	NA	NA	NA	NA	NA	NA	NA	-	NA	NA	NA	NA	NA	NA	NA	NA	NA	NA
	**13**	NA	NA	NA	NA	NA	NA	NA	NA	NA	NA	NA	-	NA	NA	NA	NA	NA	NA	NA	NA	NA
	**14**	*	*	1	*	*	1	*	*	*	NA	NA	NA	-	*	*	1	*	*	*	*	*
	**15**	*	*	*	*	2	*	2	*	*	NA	NA	NA	*	-	2	*	*	*	*	*	*
	**16-19**	*	2	1	*	*	1	*	1	*	NA	NA	NA	*	2	-	1	1	*	2	*	1
	**20**	2	2	*	2	1	*	1	2	1	NA	NA	NA	1	*	1	-	*	1	2	*	*
	**21-22**	*	*	1	*	2	*	2	*	*	NA	NA	NA	*	*	1	*	-	*	*	*	*
	**23**	*	*	1	*	*	2	*	*	*	NA	NA	NA	*	*	*	1	*	-	*	*	*
	**24**	*	*	1	*	*	2	*	*	*	NA	NA	NA	*	*	2	2	*	*	-	*	*
	**25**	*	*	*	*	2	*	*	*	*	NA	NA	NA	*	*	*	*	*	*	*	-	*
	**26-27**	*	*	1	*	2	*	2	*	2	NA	NA	NA	*	*	1	*	*	*	*	*	-

Note:1. Significantly different at p < 0.001

2. Significantly Different at p <= 0.05

* Not significantly different

NA Comparison cannot be made due to lack of data, i.e. amount of plaque

**Table 4:** Comparison of HU-criteria for fibrous plaque between hierarchical-classified groups.

		**Group**																					
		**1**	**2**	**3**	**4**	**5-6**	**7**	**8**	**9**	**10**	**11**	**12**	**13**	**14**	**15**	**16-19**	**20**	**21**	**22**	**23**	**24**	**25**	**26-27**
**Group**	**1**	-	2	1	*	*	2	*	*	*	NA	NA	NA	*	*	1	*	*	*	2	*	2	*
	**2**	2	-	1	*	*	*	*	2	*	NA	NA	NA	*	*	1	*	*	*	*	*	*	*
	**3**	1	1	-	*	*	2	2	1	1	NA	NA	NA	1	*	1	1	2	2	2	1	*	2
	**4**	*	*	*	-	*	*	*	*	*	NA	NA	NA	*	*	2	*	*	*	*	*	*	*
	**5-6**	*	*	*	*	-	*	*	*	*	NA	NA	NA	*	*	2	*	*	*	*	*	*	*
	**7**	2	*	2	*	*	-	*	1	*	NA	NA	NA	*	*	1	*	*	*	*	*	*	*
	**8**	*	*	2	*	*	*	-	*	*	NA	NA	NA	*	*	*	*	*	*	*	*	*	*
	**9**	*	2	1	*	*	1	*	-	*	NA	NA	NA	*	*	1	*	2	*	2	*	*	2
	**10**	*	*	1	*	*	*	*	*	-	NA	NA	NA	*	*	1	*	*	*	*	*	*	*
	**11**	NA	NA	NA	NA	NA	NA	NA	NA	NA	-	NA	NA	NA	NA	NA	NA	NA	NA	NA	NA	NA	NA
	**12**	NA	NA	NA	NA	NA	NA	NA	NA	NA	NA	-	NA	NA	NA	NA	NA	NA	NA	NA	NA	NA	NA
	**13**	NA	NA	NA	NA	NA	NA	NA	NA	NA	NA	NA	-	NA	NA	NA	NA	NA	NA	NA	NA	NA	NA
	**14**	*	*	1	*	*	*	*	*	*	NA	NA	NA	-	*	1	*	*	*	*	*	*	*
	**15**	*	*	*	*	*	*	*	*	*	NA	NA	NA	*	-	1	*	*	*	*	*	*	*
	**16-19**	1	1	1	2	2	1	*	1	1	NA	NA	NA	1	1	-	1	1	*	1	1	1	1
	**20**	*	*	1	*	*	*	*	*	*	NA	NA	NA	*	*	1	-	*	*	*	*	*	*
	**21**	*	*	2	*	*	*	*	2	*	NA	NA	NA	*	*	1	*	-	*	*	*	*	*
	**22**	*	*	2	*	*	*	*	*	*	NA	NA	NA	*	*	*	*	*	-	*	*	*	*
	**23**	2	*	2	*	*	*	*	2	*	NA	NA	NA	*	*	1	*	*	*	-	*	*	*
	**24**	*	*	1	*	*	*	*	*	*	NA	NA	NA	*	*	1	*	*	*	*	-	*	*
	**25**	2	*	*	*	*	*	*	*	*	NA	NA	NA	*	*	1	*	*	*	*	*	-	*
	**26-27**	*	*	2	*	*	*	*	2	*	NA	NA	NA	*	*	1	*	*	*	*	*	*	-

Note:1. Significantly different at p < 0.001

2. Significantly different at p <= 0.05

* Not significantly different

NA Comparison cannot be made due to lack of data, i.e. amount of plaque

## Discussion

Plaque rupture has been identified as the most prevalent feature at sudden coronary death cases [9]. A thin fibrous cap (<65µm) and a relatively large lipid-rich content are associated to plaque’s vulnerability to rupture [47]. Also, plaques showing positive remodeling are reported to contain more lipid-rich components [48,49]. It has been suggested that MDCT should be able to measure plaque volume [50], to detect and measure positive remodeling [51] and to even follow the change of plaque characteristics after lipid-lowering therapy [52,53], using a simple HU-based approach. Patients having low attenuation value coronary plaques as detected by MDCT were shown to be at higher risk of an acute coronary syndrome (ACS) [54].

Direct use of HU-criteria to quantify lipid-rich plaque is not trivial as considerable variability exists in the reported HU values of lipid plaque. Over the years, CT technology has advanced rapidly from producing a thick slab image during a rather long scan time to producing submillimeter thin images in subsecond scan time, allowing for accurate coronary imaging. Due to its calibration, HU value of a material or a tissue should be equal irrespective of how or with which CT system it was acquired. However, it is advised to be extra cautious in applying absolute HU-criteria when characterizing plaques as CT attenuation values were found to differ in case of different reconstruction settings [14,55]. The present study identified 36 publications, published between 1998 and 2011, each giving HU-criteria for lipid-rich and fibrous plaque. Specific patterns were found when the HU-criteria were pooled according to the reporting studies’ characteristics. Both HU-criteria for lipid-rich and fibrous plaques were significantly lower for ex-vivo studies compared to in-vivo studies, presumably due to lack of movement during scanning. HU-criteria of coronary lipid-rich plaques were significantly higher than those of other arteries (carotid and popliteal arteries). This may be caused by more partial volume effect from the surrounding fibrous tissue and lumen contrast-enhancement due to smaller plaque size and more movement during scanning. The specific way in which each CT-vendor processes scan data may cause the significant differences in HU-criteria for different CT-systems. HU-criteria for lipid-rich plaques decreased as the number of detector-rows increased (except for low HU-value of the 32-detector rows group, which is explainable because the studies did not use contrast material in the experiment) and the lipid-rich HU-criteria from the largest collimation-widths are significantly higher than the rest. This might be explained by the fact that improvements in scanner technology with higher spatial resolution result in less partial volume effect, especially from the lumen contrast-enhancement. Materials’ x-ray attenuation values depend on the x-ray photon energy, a principle behind the material decomposition with dual energy CT [56]. Our results concurred with this fact by showing that the HU-criteria for both lipid-rich and fibrous plaques were significantly higher for studies using 120 kV voltage settings than those using higher voltage settings. The significantly lower HU-criteria for studies using lower than 120 kV voltage settings is caused by the fact that the scan was performed without lumen contrast-enhancement. Lumen contrast-enhancement is one of the most frequently identified influencing sources to the non-calcified plaques’ HU value [15,57–60]. However, no direct correlation between the reported lumen contrast-enhancements and plaque HU-criteria were found in this study result. Besides by the different characteristics of the reporting studies, this lack of association may also be explained by one aspect of the measurement, i.e. the distance of the measurement ROI from the lumen border, which has been reported to affect plaque HU values [15]. Unfortunately, none of the analyzed publications reported this particular information on ROI placement which prohibits further analysis. Potential differences in patient characteristics or in tube current were not analyzed. Patient characteristics may influence the composition of plaque [47,61]. However, it should not have affected the HU value of the plaque as such. The tube current will mainly affect image quality and not the HU value of the plaque.

The investigated HU-criteria in this study were of the lipid-rich and fibrous plaque. However, more complex division of tissues is attributed to non-calcified plaque. The American Heart Association (AHA) has classified the atherosclerotic plaque into 6 types according to its composition, progression, and complexity [62,63]. In several of the included CT studies, non-calcified plaque has been characterized according to the AHA classification [32,35,36,40]. One study managed to characterize one other type of tissue, i.e. hemorrhage, on carotid atherosclerotic plaques [7]. However, due to the limitation of CT in spatial resolution to characterize each individual plaque component, most of the studies characterize non-calcified plaque into two categories only: low and high attenuation value, of which the previous attributed to lipid-rich plaque and the latter to fibrous plaque. Even then, the HU-criteria of lipid-rich and fibrous plaque still overlap largely. Some of the included studies proposed a HU-threshold or -range to characterize different plaque components [7,17,19,22,23,25,29,30,34,39,42]. Receiver operating characteristic (ROC) analysis was used to determine some threshold, showing promising accuracies (sensitivity ranged from 82% to 92%) [19,25,29,30,42].

An HU-based plaque characterization approach was used to quantify non-calcified plaques in patients in a number of studies. Some studies applied the HU-criteria published by their own group and thus with exact study characteristics match [54,64,65]; while another group applying HU-criteria of group other than theirs but with matching study characteristics [66]. However, sometimes, HU-criteria coming from studies with different characteristics than their own were applied, ranging from small differences, e.g. the generation of the CT-system used [67], to larger differences, e.g. the brand and detector-rows [53,68]; the brand and vessel type [69]. The original studies that published the HU-criteria and test them have shown promising accuracies. This is presumably caused by the fact that the used HU-criteria match the study characteristics exactly. As has been shown in the present study, HU-criteria for non-calcified plaque derived from studies with different study characteristics may be significantly different. This could result in considerably different measurements of non-calcified plaque components. Since obtaining the correct amount of lipid-rich plaques is of importance in determining the extent of vulnerable plaque [9], it is theoretically preferable to characterize plaques using criteria which match one’s specific study characteristics.

Some reviews exist on non-calcified plaque characterization by CT [70,71]. We managed to extend the discussion by systematically investigating the published HU-criteria based on specific characteristics of each study. By hierarchically grouping the HU-criteria based on the study characteristics, the effect of each characteristic was separately analyzed. As a result, 21 and 22 distinct HU-criteria were obtained from lipid-rich and fibrous plaque, respectively. Two post-mortem studies with histopathological correlation and an in-vitro validation study without reference standard reported non-significant differences in HU-criteria when using different CT-system brands [46] and voltage-settings [44,46,72]. In our study, some of the pooled and grouped HU-criteria comparisons were also not significantly different, but a significant portion of the comparisons were, indicating that specific HU-criteria correspond to specific characteristics of each study. This warrants a careful selection of the HU-criteria, should non-calcified plaque characterization be desired. Therefore, the proposed hierarchical diagram may be consulted for using the HU-criteria in clinical practice (Figures 3 and 4). The most suitable HU-criteria for lipid and fibrous plaque for a specific clinical situation can be traced, e.g. when an in-vivo examination of coronary plaque should be done on a Siemens 64-row MDCT at 120 kV, the HU-criteria of group 9 in the diagram should be used.

A limitation of our study is that the provided HU-criteria hierarchical diagram is not 100% complete as not every combination of characteristics is currently available in literature. Moreover, with the advent of more advanced CT-systems, such as the 320-MDCT and 0.23 mm spatial resolution, new HU-criteria can emerge. Not every HU-criterion presented in the diagram has the same accuracy due to unequal number of supporting studies or samples and therefore, clinical application still leaves room for improvements. Several limitations can be traced into possible biases in the included study. First, since all the included studies are published, publication bias cannot be ruled out. Most of the included studies are on English, but no language preference is set for the selection criteria. The number of data in each included studies varies considerably. In fact, several studies have a very small number of data that we can suspect also the possibility of small sample bias. However, for the sake of completeness of the diagram, the results from those small sample studies are included in the diagram. The hierarchical diagram is aimed to provide a guidance to seek the most suitable HU-criteria for the local settings. The number of the studies and their analyzed samples are reported also in the diagram. This information should warn the user of the diagram on the potential bias it contains. Further research should provide more input for the proposed diagrams involving phantoms or arterial specimens with known plaque compositions scanned using multiple CT-systems at different settings and a clinical validation to establish a clinically useful guide with which HU-criteria can be applied per study set-up.

## Conclusions

Criteria to characterize non-calcified plaques based on CT attenuation value are non-uniform, due to differences in examination type, vessels of interest, and CT scanning. Therefore, generalized values are unreliable for clinical use. The proposed hierarchical classification can be used to determine reference CT attenuation value values of lipid-rich and fibrous plaques for the local setting.

## Supporting Information

Table S1 **PRISMA Checklist.**
(DOC)
